# Pulmonary Mycetoma With a Concomitant Reactivation of Pulmonary Tuberculosis Infection: A Case Report and Clinical Pathological Review

**DOI:** 10.7759/cureus.35459

**Published:** 2023-02-25

**Authors:** Wendolin J Ortiz, Robert L McKowen, Mario Cervantes

**Affiliations:** 1 Pathology, HCA (Hospital Corporation of America) Houston Healthcare West, Houston, USA; 2 General Surgery, Universidad Autónoma de Baja California, Mexicali, MEX; 3 Cardiothoracic Surgery, HCA (Hospital Corporation of America) Houston Healthcare West, Houston, USA; 4 Pathology, HCA (Hospital Corporation of America) Houston Healthcare Pearland, Houston, USA

**Keywords:** pulmonary cavitation, pulmonary aspergillosis, concomitant infection, mycetoma, tuberculosis

## Abstract

Tuberculosis is a bacterial infection caused by *Mycobacterium tuberculosis*. It primarily affects the lungs but can also spread to other body parts. One of the possible symptoms of pulmonary tuberculosis (TB) is hemoptysis. In the case of TB, aspergillomas can develop in the cavitary lesions of TB and result in a deteriorating clinical situation. The current case report describes a 63-year-old female previously treated for TB who presented with hemoptysis, fever, and a 4 cm focal density in the right upper lobe on chest X-ray. The patient was found to have concomitant TB and aspergillosis, manifesting as a pulmonary aspergilloma. The co-occurrence of TB and aspergillosis can occur, particularly in patients with weakened immune systems. This case report highlights the importance of considering concomitant TB and pulmonary mycetoma in patients with a history of treated TB who present with pulmonary symptoms.

## Introduction

Hemoptysis is a common symptom of both pulmonary mycetoma and reactivated pulmonary tuberculosis (TB) infection. A fungal ball, also known as a mycetoma, can be caused by different fungi, including Aspergillus species. It is a rare condition in which Aspergillus fungi form clumps in the tissue, typically in the lungs or sinuses, leading to chronic inflammation and producing blood-tinged sputum [1]. On the other hand, reactivated TB infection is common in individuals who have previously been treated for TB but have not completed the course of antibiotics; common symptoms include persistent cough, hemoptysis, chest pain, fatigue, fever, night sweats, and weight loss [2].

Aspergillosis is commonly found to co-occur with TB in immunocompromised patients, with a prevalence of up to 80% in some studies [3-5]. A case of the simultaneous presence of both mycetoma and TB infection occurring in an immunocompetent individual was recently reported [6]. The presence of both TB and aspergillosis can result in an increased risk of hemoptysis compared to either infection alone; life-threatening hemoptysis is best managed with early surgical intervention, especially in cases where aspergilloma or other cavitary lesions are present [2,7,8].

## Case presentation

**Figure 1:**
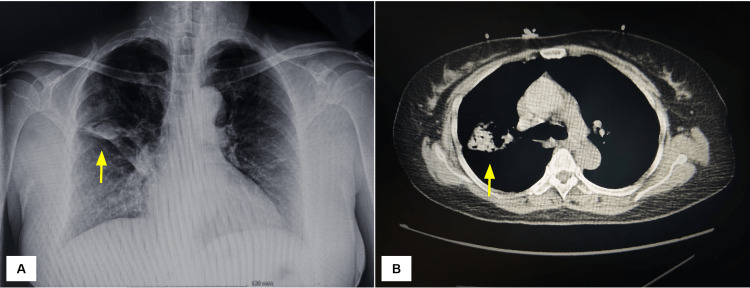
Chest imaging findings (A) Anterior-posterior chest radiograph showing a focal density in the right upper lobe with adjacent linear scarring/atelectasis and (B) CT scan of the chest showing a partial consolidation of the right anterior and posterior segments of the right upper lobe. CT: computed tomography.

A 63-year-old woman with a history of diabetes mellitus type 2, hypertension, and coronary artery disease presented to the emergency room with a complaint of hemoptysis that had persisted for over a month; she had previously been treated for TB. The patient was planning to visit the USA, and the direct acid-fast bacilli (AFB) test was negative during her pre-travel check-up. After reaching here, she started having fever and hemoptysis, which had become more frequent. An X-ray in the emergency room showed a focal 4 cm density in the right upper lobe with adjacent linear scarring and atelectasis. A chest computed tomography (CT) scan confirmed a partial consolidation of the right upper lobe's anterior and posterior segments, with a 2 cm ovoid region of relatively low attenuation and without a crescent sign. There was an adjacent tree in bud with nodular opacities in the posterior segment of the right upper lobe. A linear consolidation in the medial aspect of the right middle lobe and linear atelectasis or scarring in the lingula were seen (Figure 1).

The patient was admitted for management with cardiovascular surgery and pulmonary services; she stated having occasional chest pain in the right upper chest with a cough but nothing excruciating, no shortness of breath but presented wheezing at times. Oral thromboxane in the dose of 500 mg every four hours, as needed for active hemoptysis, was given. After a week of observation, she was clear of any phlegm or bleeding and was considered stable for surgery. Cardiovascular surgery did the exploration; the right pleural space was entered, and the patient was found to have severe, dense right pleural adhesions; the entire lung was encased in dense pleural adhesions, most likely from previous infections, which took a significant amount of time to clear. With the lung completely freed up from the thoracic wall, diaphragm, and mediastinum, the bilobectomy of the right upper and middle lobes was performed.

**Figure 2:**
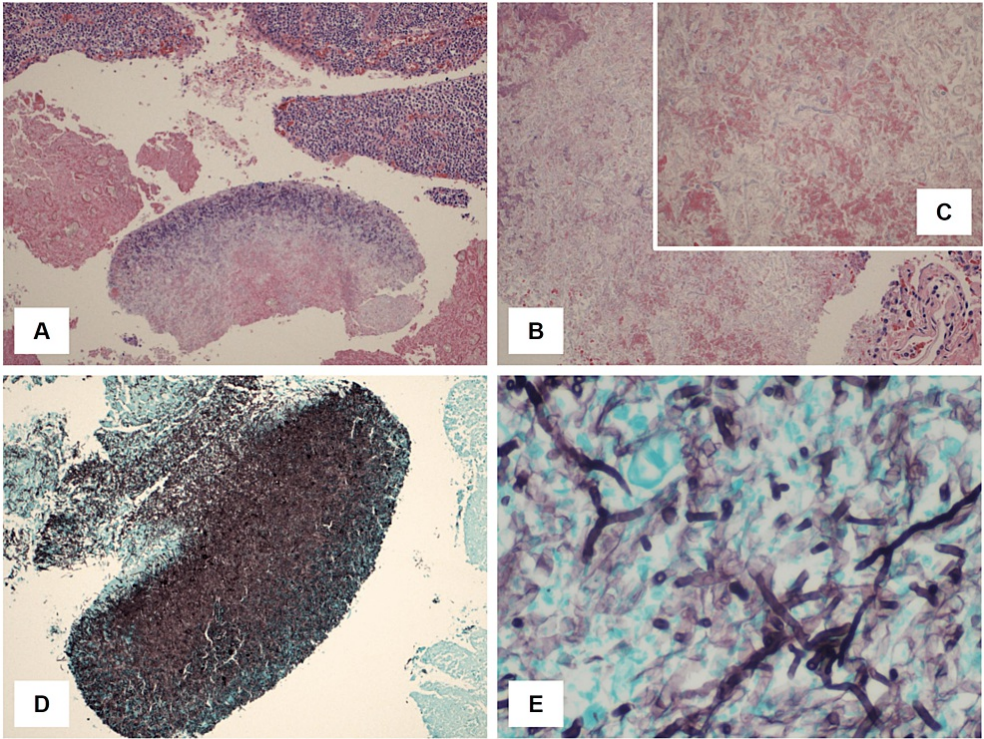
Microscopic images of mycetoma and fungal organisms associated with aspergillosis (A) A low-power view of the mycetoma with adjacent respiratory epithelium (H&E, 100×); (B) a higher magnification view of the fungal ball (H&E, 200×); (C) a close-up view of the mycetoma showing numerous fungal organisms (H&E, 600×); (D) the fungal ball stained with Gomori Methenamine Silver (GMS) showing fungal structures (100×); (E) the Aspergillus fungal ball stained with GMS showing branching fungal hyphae (400×). H&E: hematoxylin and eosin.

**Figure 3:**
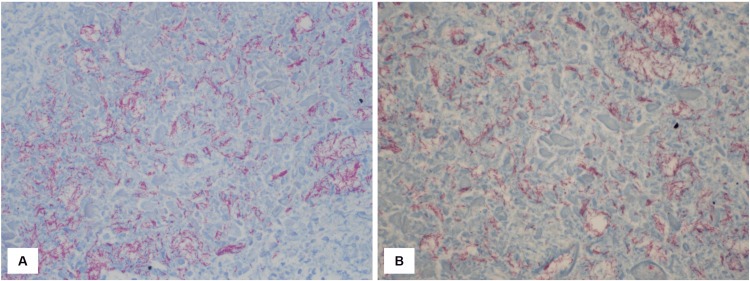
Microscopic images of acid-fast bacilli (AFB) (A) Acid-fast bacilli stain positive with abundant bacteria at 200× magnification and (B) a higher magnification view (600×) showing numerous AFB.

Received in the pathology department was a segment of the lung measuring 17.5 cm × 11 cm × 4 cm with a purple, smooth, and vascular pleural surface. The cut section revealed a mass measuring 3.5 cm × 3 cm × 2.7 cm, located 2.6 cm from the bronchial margin. Additional sectioning of the lung revealed areas of calcified tan-white plaque and a cyst filled with brown hemorrhagic content measuring 2.2 cm × 1.8 cm × 1.5 cm in the upper lobe, along with two anthracotic pigment nodes that measured 1.2 cm × 1.5 cm. The histopathological examination revealed the presence of TB (AFB stained positive with abundant bacteria), acute and chronic bronchopneumonia with partly organized fibrosis and associated hemorrhage (acute and chronic), bronchiectasis with Aspergillus fungal balls (Gomori Methenamine Silver (GMS) stained positive), caseating small granulomas with associated fibrosis, and prominent anthracosis were also present (Figures 2-3).

Following the surgery, the patient's recovery was uneventful. She was successfully extubated and showed signs of improvement in subsequent chest X-rays with decreasing air leak and minimal bleeding. After nine days of being discharged from the intensive care unit (ICU), the patient experienced a sudden change in condition, suffering from a severe bout of hemoptysis. Despite being transferred back to the ICU and receiving resuscitative measures, the patient's oxygen levels declined, and unfortunately, she passed away due to continued bleeding.

## Discussion

Hemoptysis is a serious medical condition characterized by coughing up blood and usually caused by underlying inflammatory processes. In our case, a 63-year-old female patient had been treated for TB in her home country, and with recent negative sputum AFB, she was found to have reactivation of TB and a cavitary lesion in her right upper lobe, which led to the development of a pulmonary aspergilloma and the onset of hemoptysis. Given the severity of her condition, surgical resection was recommended, as medical treatment alone would not suffice.

TB is a respiratory infection caused by *Mycobacterium tuberculosis*; it is one of the leading causes of death worldwide, with high incidence in countries like India, Pakistan, sub-Saharan Africa, South Africa, Eastern Europe, and China, and is spread by close person-to-person contact through the inhalation of infectious aerosols. *M. tuberculosis* is an intracellular parasite that invades the respiratory airways and infects alveolar macrophages. It prevents phagosome-lysosome fusion, leading to intracellular replication and the evasion of macrophage killing. Macrophages respond to the infection by secreting cytokines like interleukin (IL)-12 and tumor necrosis factor (TNF)-a, leading to increased inflammation and T-cell differentiation into TH1 cells. These activated macrophages, with the help of interferon (IFN)-y, lead to increased phagosome-lysosome fusion and intracellular killing [2,4,5,7,9]

The symptoms of TB reflect the site of infection, with the primary disease usually restricted to the lower respiratory tract. Common symptoms include malaise, weight loss, cough, night sweats, and hemoptysis. The diagnosis is supported by radiographic evidence, a positive skin test, and laboratory detection of *M. tuberculosis*. The tuberculin skin test is the traditional test to assess the patient's response to exposure to *M. tuberculosis*. However, its efficacy is limited in regions where the Bacillus Calmette-Guerin (BCG) vaccine is widespread. Microscopic detection of acid-fast bacteria in clinical specimens is a quicker way to confirm mycobacterial disease [9].

Most TB treatment regimens involve a combination of drugs like isoniazid (INH), ethambutol, pyrazinamide, and rifampin for two months, followed by four to six months of INH and rifampin or alternative combination drugs. The BCG vaccine is commonly used in countries where TB is endemic; patients who do not respond to drug therapy are candidates for surgery. In infected patients, the surgical specimens show inflammation, fibrosis, and non-functioning lung parenchyma, associated with bronchiectasis, atelectasis, and superimposed infections [1,9]. Chronic pulmonary aspergillosis is commonly reported as a sequel to TB [8]. In this case, the patient was previously treated for TB in her home country and had a reactivation of the TB infection, which also resulted in the development of a pulmonary aspergilloma.

Aspergillosis is a fungal infection caused by Aspergillus spp. conidia that can settle in the lungs, nasopharynx, or sinuses via inhalation. The severity of the disease and the resulting symptoms depend more on host factors than on the virulence of the fungus. Aspergillosis can lead to airway colonization, allergic reactions, or the colonization of pre-existing cavities, leading to the development of an aspergilloma, which is a ball-like structure composed of fungal elements, fibrin, mucus, and cellular debris and may be asymptomatic or cause fever, pulmonary infiltrates, chest pain, and hemoptysis. The diagnosis is usually confirmed by laboratory testing and radiology, and treatment options depend on the severity of the infection, ranging from antifungal drugs to surgical resection. Surgical resection may be necessary in severe cases of pulmonary hemorrhage. The infection can also disseminate to extrapulmonary sites due to the angioinvasive nature of the fungus. Despite specific antifungal therapy, the mortality rate of this infection is high and usually exceeds 70% [9].

The patient's history of previously treated TB and the presence of *M. tuberculosis* in the pathological examination support the reactivation of the TB infection. Chronic pulmonary aspergillosis commonly complicates treated TB with residual cavitation [5]; a systematic review and meta-analysis of cross-sectional studies from Asia and Africa provide further evidence of the high prevalence of Aspergillus coinfection among immunocompromised patients with TB [7]. The current case highlights the presence of concomitant TB and a pulmonary mycetoma in an immunocompetent host, which is a rare occurrence [6]. Further research is necessary to better understand this co-infection's epidemiology and clinical characteristics, especially in high TB-burden countries, to guide clinical management and improve patient care.

## Conclusions

The co-occurrence of TB and aspergillosis is a rare event. Hemoptysis is a common symptom of both pulmonary mycetoma and reactivated TB, and the presence of both infections can increase the risk of hemoptysis. In this case, a multidisciplinary team of specialists managed the patient's condition, with surgical resection being the preferred option. Treatment of these infections usually involves a combination of drugs and, in some cases, surgery, necessitating close monitoring and follow-up to ensure a full recovery. Early diagnosis and treatment of this unusual occurrence are crucial in preventing morbidity and mortality.
